# Genome wide association studies for acid phosphatase activity at varying phosphorous levels in *Brassica juncea* L

**DOI:** 10.3389/fpls.2022.1056028

**Published:** 2022-12-20

**Authors:** Priyanka Upadhyay, Mehak Gupta, Simarjeet Kaur Sra, Rakesh Sharda, Sanjula Sharma, Virender K. Sardana, Javed Akhatar, Gurpreet Kaur

**Affiliations:** ^1^ Department of Plant Breeding and Genetics, Punjab Agricultural University, Ludhiana, India; ^2^ Department of Soil & Water Engineering, Punjab Agricultural University, Ludhiana, India

**Keywords:** Indian mustard, acid phosphatase, phosphorus use efficiency, SNP genotyping, marker trait associations

## Abstract

Acid phosphatases (Apases) are an important group of enzymes that hydrolyze soil and plant phosphoesters and anhydrides to release Pi (inorganic phosphate) for plant acquisition. Their activity is strongly correlated to the phosphorus use efficiency (PUE) of plants. Indian mustard (*Brassica juncea* L. Czern & Coss) is a major oilseed crop that also provides protein for the animal feed industry. It exhibits low PUE. Understanding the genetics of PUE and its component traits, especially Apase activity, will help to reduce Pi fertilizer application in the crop. In the present study, we evaluated 280 genotypes of the diversity fixed foundation set of Indian mustard for Apase activity in the root (RApase) and leaf (LApase) tissues at three- low (5µM), normal (250µM) and high (1mM) Pi levels in a hydroponic system. Substantial effects of genotype and Pi level were observed for Apase activity in both tissues of the evaluated lines. Low Pi stress induced higher mean RApase and LApase activities. However, mean LApase activity was relatively more than mean RApase at all three Pi levels. JM06016, IM70 and Kranti were identified as promising genotypes with higher LApase activity and increased R/S at low Pi. Genome-wide association study revealed 10 and 4 genomic regions associated with RApase and LApase, respectively. Annotation of genomic regions in the vicinity of peak associated SNPs allowed prediction of 15 candidates, including genes encoding different family members of the acid phosphatase such as *PAP10* (purple acid phosphatase 10), *PAP16*, *PNP* (polynucleotide phosphorylase) and *AT5G51260* (HAD superfamily gene, subfamily IIIB acid phosphatase) genes. Our studies provide an understanding of molecular mechanism of the Apase response of *B. juncea* at varying Pi levels. The identified SNPs and candidate genes will support marker-assisted breeding program for improving PUE in Indian mustard. This will redeem the crop with enhanced productivity under restricted Pi reserves and degrading agro-environments.

## 1 Introduction

Phosphorus (P) is one of the most crucial macronutrients required for the optimal growth and development of plants (Han et al., 2022; Bhadouria and Giri, 2022). This element is a prime structural constituent of cell biomolecules, including ATP, NADPH, phospholipids, and nucleic acids (Kohli et al., 2020). Significant yield losses in cereal (Plenet et al., 2000; Wissuwa and Ae, 2001; Lazaro et al., 2010), pulse (Bonser et al., 1996; Mahamood et al., 2009), fodder (Camacho et al., 2002; Ceasar et al., 2014 and oilseed crops (Bastani and Hajiboland, 2017; Grzebisz et al., 2018) have been reported under P deficient conditions (Maharajan et al., 2018). Surprisingly, majority (71%) of the global cropland area has a surplus of P, whereas 29% is in a state of P deficiency (Thudi et al., 2021). However, soils that carry ample P to support plant growth also tend to show P deficiency (Raghothama and Karthikeyan, 2005). It is due to the low (1-10 µM) availability of soluble inorganic phosphate (Pi; H_2_PO_4_^−^ or HPO_4_^2−^), the only forms that plants can absorb and assimilate (Shen et al., 2011). Pi has high propensity to form insoluble complexes with metal cations such as aluminum and iron in acidic soils and calcium and magnesium in alkaline soils, which render Pi unavailable to plants (Bhadouria and Giri, 2022). Also, an abundant amount of P (30–80% of total P) remains fixed in soil as Po (organophosphate) and becomes unavailable for root acquisition unless hydrolyzed by enzymes to liberate absorbable Pi (Richardson and Simpson, 2011; Dissanayaka et al., 2018). Phosphate fertilizers are routinely applied to alleviate Pi deficiency and maintain crop yields and quality. It is estimated that there is a requirement for 51–86% more Pi inputs by 2050 to sustain global food production (Mogollon et al., 2018). At the same time, phosphate rock, a non-renewable Pi resource, is diminishing at a faster rate and may be completely depleted in the next 100–400 years (Gilbert et al., 2009; Desmidt et al., 2015). Furthermore, excessive use of inorganic fertilizers has led to potential environmental problems as most of the applied Pi is not recovered by crops (Syers et al., 2008). Most crop plants absorb less than 20% of applied Pi (Plaxton and Tran, 2011). A significant amount of Pi coprecipitates and may run off from the soil to surface waters, resulting in aquatic eutrophication (Zak et al., 2018). In view of these concerns, there is a need to divert efforts towards engineering crop cultivars that acquire and utilize Pi more efficiently to produce higher yields under Pi limited conditions (Cong et al., 2020).

Plants elicit a series of alterations at morphological, physio-chemical and molecular levels such as changes in root architecture, root to shoot ratio, membrane structure, anthocyanin accumulation, secretion of organic acids (malate, citrate, oxalate etc.) and hydrolases (phospholipases, ribonucleases, acid phosphatases etc.) and activation of various Pi stress response genes to increase Pi acquisition and utilization to sustain plant growth under Pi limited conditions (Plaxton and Tran, 2011; Ryan et al., 2014; Li et al., 2016). Collectively, these response mechanisms induced to maintain plant Pi homeostasis are known as Pi starvation response (PSR). Amongst these adaptive responses, induction and secretion of acid phosphatase (Apase) enzymes is a universal response. They catalyze hydrolysis of organic P complexes (phosphoesters and anhydrides) in acidic soils and plant tissues to release soluble Pi for plant utilization (Raghothama, 1999; Nannipieri et al., 2011; Gu et al., 2016). Significant positive correlations between Apase activity and PUE (phosphorus use efficiency) have been recorded (Chen et al., 2003; Radersma and Grierson, 2004; Zhang et al., 2010). In rice, overexpression of Apase genes (*OsPAP10a, OsPAP10c, and OsPAP21b*) significantly increased the hydrolysis and utilization of externally supplied Po and ATP under low Pi conditions (Tian et al., 2012; Lu et al., 2016; Mehra et al., 2017; Deng et al., 2020). Thus, genetic manipulation of Apase activity is of high interest for improving the PUE of plants (Han et al., 2022). Purple acid phosphatases (PAPs) are the most studied class of Apases in relation to P homeostasis. To date, the identification of PAPs has been completed for several plant species (Bhadouria and Giri, 2022). They are also known to be present in bacteria and animals, executing a similar function (Wang et al., 2021; Bhadouria and Giri, 2022). In plants, PAPs occur as a multigene family. There are 29 members of PAPs in *Arabidopsis thaliana*, 26 in *Oryza sativa*, 33 in *Zea mays ssp. mays* var. B73, 38 in *Glycine max*, 25 in *Cicer arietinum*, 19 in *Camellia sinensis* and 25 in *Jatropha curcas* (Li et al., 2002; Zhang et al., 2011; Li et al., 2012; Gonzalez-Munoz et al., 2015; Bhadouria et al., 2017; Venkidasamy et al., 2019; Yin et al., 2019; Srivastava et al., 2020). In contrast, other Apases such as halogenated acid dehalogenase (HAD), polynucleotide phosphorylase (PNPase) and protein phosphatases (PP2C) are less investigated in plants in response to Pi variations (Marchive et al., 2010; Khan et al., 2018; Su et al., 2021; Bhadouria and Giri, 2022). The molecular regulation of Apase expression is found to be complex in nature. A number of transcription factors like *PHR1 (PHOSPHATE STARVATION RESPONSE 1*) and its homologues (*PHR-like- PHL1, PHL2*, and *PHL3*), *WRKY75, ZAT6 (ZINC FINGER OF ARABIDOPSIS THALIANA 6), OsMYB2P-1 (Oryza sativa MYB2 phosphate-responsive gene 1), StMYB44 (Solanum tuberosum MYB transcription factor)*, and *AP2/ERF (APETALA 2/ethylene-responsive element binding factor*) regulate Apase activity (Dai et al., 2012; Wang et al., 2013; Zhou et al., 2017). SPX proteins (*SYG1, PHO81* and *Xpr1*) indirectly control PAP activity by binding to PHR/PHL transcription factors. In monocots, SPX proteins show low affinity to PHR/PHL transcription factors, thus inducing PAPs under Pi deficient conditions (Wang et al., 2014; Puga et al., 2014). The trend is opposite in dicots, where PAPs are positively regulated by SPX proteins. In addition, phytohormones (auxins, cytokinins and ethylene) and sugar signalling, miRNA399 expression, and some post-translation modifications such as glycosylation strongly influence Apase activity (Puga et al., 2017). GWAS (genome wide association study) is a classical method for studying the genetic basis of complex quantitative traits (Pal et al., 2021). It takes full advantage of historical recombination events coupled with high allelic diversity of the association panels for fine mapping of genetic loci (Rafalski, 2010; Huang and Han, 2014). Indian mustard (*Brassica juncea* L. Czern & Coss, 2n = 4x = 36, genome AABB) is an important crop species that provides oil for human consumption and protein rich extraction meal for the animal industry (Goel et al., 2018). Its yield and quality are severely affected by low Pi availability in the soil (Zhang et al., 2009; Yao et al., 2011). So, breeding mustard varieties with enhanced PUE is imperative for sustainable agriculture. Unveiling the molecular mechanisms of different players of plant adaptation to low Pi will help to design Pi efficient cultivars. In the present study, we analyzed a wide germplasm set (280 genotypes) of Indian mustard for Apase activity in root and leaf tissues at three Pi levels in a hydroponic system. GWAS enabled us to study the association of SNP markers with genetic variation for Apase activity. The identified marker-trait associations (MTAs) and candidate genes in the present investigation will support the development of P efficient cultivars *via* marker assisted breeding.

## 2 Materials and methods

### 2.1 Plant materials

A diversity fixed foundation set of 280 genotypes of *B. juncea*, including landraces, historical varieties, cultivars, resynthesized and determinate *B. juncea*, alloplasmic lines and introgression lines was evaluated for Apase activity in the root (RApase) and leaf (LApase) tissues at three Pi levels: low (LP; 5µM), normal (NP; 250µM) and high (HP; 1mM) in a hydroponic system (**Supplementary Table 1**). The diversity fixed foundation set collection was established at Punjab Agricultural University, Ludhiana, under the ICAR (Indian Council of Agricultural Research) funded NASF (National Agricultural Science Fund) project: “Creating a fully characterized genetic resources pipeline for mustard improvement”.

### 2.2 Plant growth conditions and Apase activity measurement

The hydroponic experiment was conducted twice at an experimental farm of the Department of Soil and Water Engineering, Punjab Agricultural University, Ludhiana, from July 2020 to September 2020. An in-house developed hydroponic system was deployed for the current study. For this, seven PVC pipes (length: 609.6 cm; diameter: 10 cm) were installed on an angle iron frame in a pyramidal arrangement. The bottom pipes were maintained at the height of 75 cm above the ground. 20 holes/pipe were drilled at a spacing of 30 cm to retain pots of diameter- 7.5 cm for plant growth. The growth conditions of the polyhouse were maintained at 25°/18°C day/night temperature with relative humidity of 70 ± 2%. Two seeds of uniform size from each of 280 genotypes were sown in portray, after surface sterilization in a 0.5% (w/v) sodium hypochlorite solution for 15 minutes, followed by three washings with deionized water. Virgin plasticware and glasswares were used in the whole experiment to avoid Pi contamination, if any. After seed germination, seedlings were watered with a quarter strength of modified Hoagland’s solution twice a day. The modified full-strength Hoagland’s solution was comprised of 4.5 mM Ca(NO_3_)_2_•4H_2_O, 1 mM KH_2_PO_4_, 4 mM KNO_3_, and 2 mM MgSO_4_•7H_2_O as macronutrients, and 0.32 μM CuSO_4_•5H_2_O, 46 μM H_3_BO_3_, 50 μM EDTA-Fe, 0.37 μM NaMoO_4_•2H_2_O, 9.14 μM MnCl_2_•4H_2_O and 0.77 μM ZnSO_4_•7H_2_O as micronutrients (Hoagland and Arnon, 1950). Three levels of Pi were adjusted using KH_2_PO_4_ as low (5µM), normal (250µM) and high (1mM). Under LP and NP conditions, 5 μM and 250 μM KH_2_PO_4_ were supplied along with 0.50 mM and 0.26 mM KCl respectively. Upon the emergence of true leaves, seedlings were shifted to the hydroponic system with one seedling/pot. After five days of shifting, the quarter-strength nutrient solution was progressively increased to a full-strength solution once a week until tissue sampling. The pH and EC of the nutrient solution were adjusted to 5.7 ± 0.2 and 1.5–2.5 ds/m, respectively (Greenway and Munns, 1980). Twenty-eight days old seedlings were taken for root and leaf sampling. Fresh weights of root and leaf tissues were estimated before measuring Apase activity. The standard protocol was followed to measure Apase activity in root and leaf tissue (Tabatabai and Bremner, 1969). 100 mg of root or leaf tissue was ground into a fine powder in liquid nitrogen and then homogenized in 3 ml of 0.1 M sodium acetate buffer, pH 5.0. The homogenate was centrifuged at 10,000 rpm for 15 min at 4°C. Further, 0.1 mL of the supernatant containing enzyme extract/intracellular proteins was mixed with 1.9 mL of sodium acetate buffer and 1 mL of p-nitrophenyl phosphate (p-NPP) as the substrate. Reactions proceeded for 15 min at 37°C and was terminated using one ml of 2N NaOH. p-nitrophenol (pNP) accumulation was read at 410 nm wavelength in the spectrophotometer (Techcomp UV 2600) (McLachlan et al., 1987 *)*. Apase activity was recorded as μmol of p-NP liberated min^-1^ g^-1^ fresh tissue weight (FW) from p-NPP.

### 2.3 Statistical analysis

Pooled analysis of variance was performed using Minitab v 19.0 software to assess the significance of variance due to genotype, Pi-levels, and environment, and all possible interactions between these parameters (genotype x environment, genotype x Pi-level, and Pi-level x environment) for estimated traits. Descriptive statistics and Pearson’s correlation coefficients (r) between the estimated traits were calculated using Minitab v 19.0 software.

### 2.4 Genotyping by sequencing and genome wide association study

Genotyping by sequencing data of the NASF diversity set was generated under National Agricultural Science Fund aided project “Creating a fully characterized genetic resources pipeline for mustard improvement” (Bio-Project INRP000037). The clean reads of genotypes were aligned to the reference genome of an oleiferous type of *B. juncea* variety Varuna (NCBI bioproject: PRJNA550308) using BWA software (Li and Durbin, 2009). SNP calling was then performed by employing the NGSEP-GBS (Next Generation Sequencing Experience Platform-GBS) pipeline (Duitama et al., 2014). From this marker dataset, SNPs showing minor allele frequency < 0.05, missing data >30% and heterozygosity >10% were removed. The resultant 3, 72,285 SNPs were used for GWAS analysis. BLUPs (Best linear unbiased predictions) datasets across two environments for the estimated traits (RApase and LApase) along with 3, 72,285 filtered SNPs were used as input data for GWAS analysis. BLUPs were estimated using META-R (Multi Environment Trial Analysis with Version 6.0) (https://data.cimmyt.org/dataset.xhtml?persistentId=hdl: 11529/10201) (Alvarado et al., 2020). Principal component analysis (PCA) of genotypic data was performed in R. We used different algorithms GLM (general linear model), MLM (mixed linear model), MLMM (multiple loci mixed linear model), Farm CPU (Fixed and random model Circulating Probability Unifcation) and BLINK (Bayesian-information and Linkage disequilibrium Iteratively Nested Keyway) installed in the GAPIT3 (Genome Association Predict Integrate Tools v3.0) package of R software, incorporating principal components (PCs) and kinship matrices as covariates, to execute association analysis (Wang and Zhang, 2021). Best fit algorithm was predicted using multiple Quantile-quantile (Q-Q) plots for the estimated traits. An arbitrary threshold of -log10 (P) = 3.00 was used as the suggestive threshold to term an association between SNP and trait as significant (To et al., 2019; Mai et al., 2021). The GAPIT3 package was also used to construct Manhattan plots. The genomic regions around the identified peak SNPs (50-kb upstream and 50-kb downstream of the peak SNP) were annotated to scrutinize potential candidate genes pertinent to Apase activity using Blast2GO v5.2.5 tool (Gotz et al., 2008).

## 3 Results

### 3.1 Phenotypic variation for Apase activity

**Figure 1:**
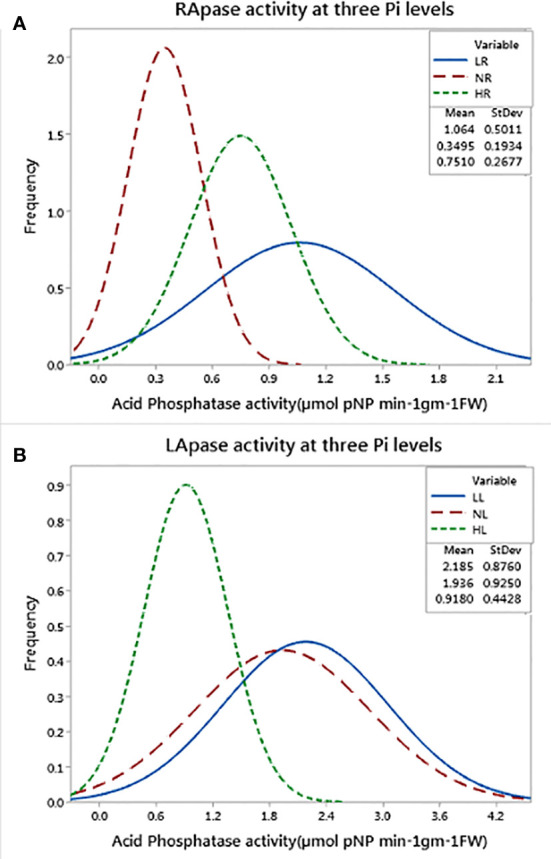
Frequency distribution of **(A)** RApase and **(B)** LApase at three Pi levels.

**Figure 2:**
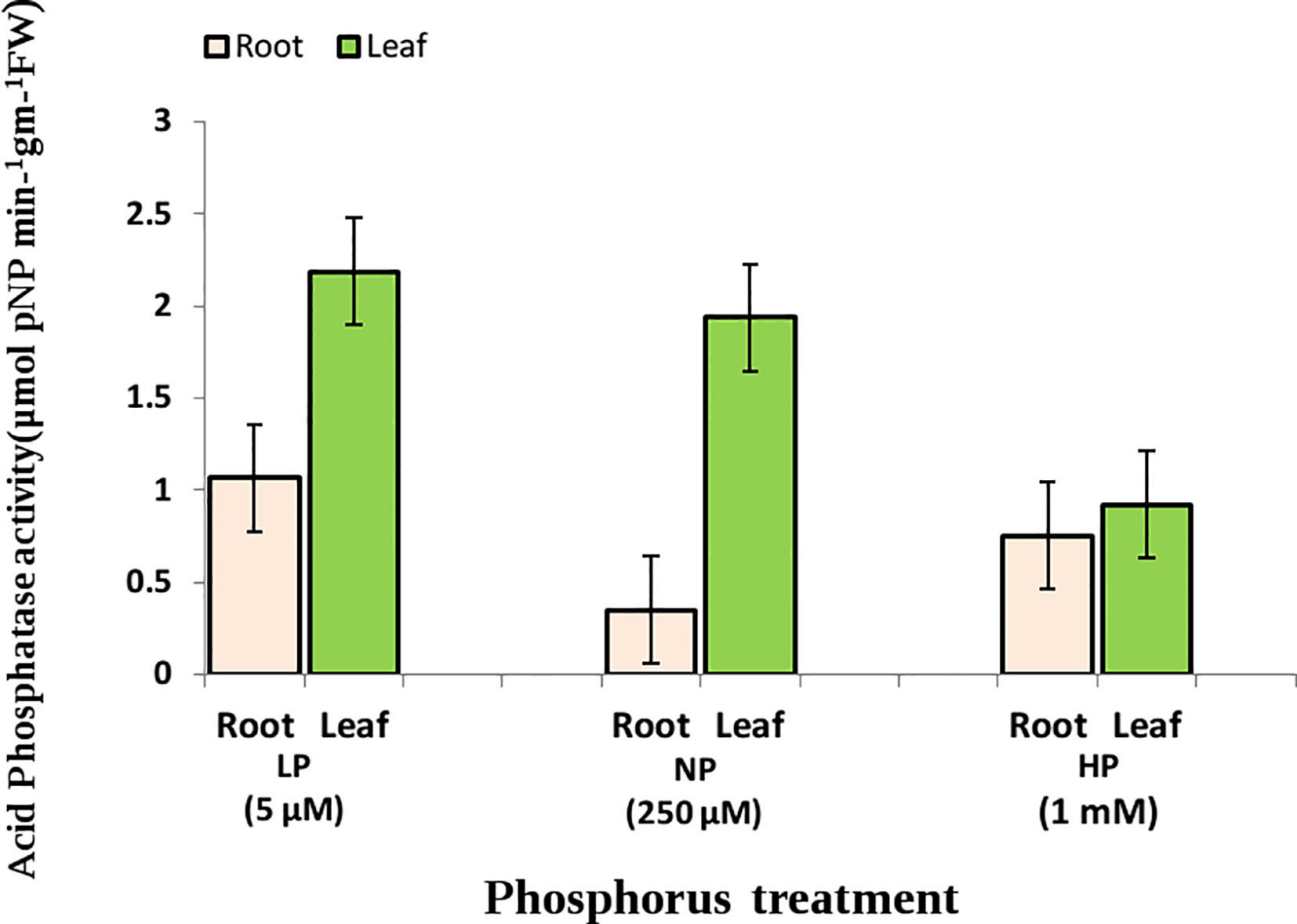
Acid phosphatase activity (in root and leaf) at three Pi levels.

Significant effects of genotype and Pi level were observed for the estimated traits (RApase, LApase and R/S) among the tested genotypes. Genotype × Pi level interactions were also significant, whereas genotype × environment was found to be non-significant (**Table 1**). Traits showed near normal distribution at all three Pi levels, with variations across levels (**Figure 1**). Descriptive statistics of the examined traits under three Pi levels are given in **Table 2** and **Figure 2**. Low Pi induced higher Apase activity by 67% and 29% in root and 11% and 58% in leaf tissues as compared to NP and HP, respectively. However, LApase exhibited a comparatively higher mean value than RApase by 51%, 82% and 18% at LP, NP and HP levels respectively. The mean LApase ranged from 0.05 to 4.31, 0.03 to 4.24 and 0.02 to 1.93 µmole p-NP min^-1^g^-1^FW in LP, NP and HP applications, respectively. Genotypes Pusa-Mahak, JM-06016, IM-170 and Kranti were found with high LApase activity (>3.6 µmole p-NP min^-1^g^-1^FW) at low Pi dose. Mean RApase ranged from 0.15 to 2.19, 0.05 to 0.83 and 0.05 to 1.14 µmole p-NP min^-1^g^-1^FW in LP, NP and HP doses, respectively. Genotypes RB-50, RH-9308-1, IM-127 and JT-1 possessed high RApase values (> 2.1 µmole p-NP min^-1^g^-1^FW) at low Pi supply. The R/S increased significantly by 52% and 56% at LP in comparison to NP and HP, respectively. IM-170, Kranti, JM-06016 and JT-1 were the promising genotypes, depicting higher Apase activity and increased R/S ratio under LP condition. IM-170, Kranti and JM-06016 showed greater LApase, while JT-1 was identified with higher RApase activity. The coefficient of variation (CV) was highest for RApase at NP (55.34) followed by LApase at HP (48.23%) and NP (47.78%). Pairwise Pearson’s correlation coefficients revealed a negative correlation between LApase and RApase at HP (P<0.05), while at LP and NP level they exhibited no correlation to each other (**Table 3**). The R/S exhibited a positive correlation with LApase under LP condition.

**Table 1:** Analysis of variance for RApase, LApase and R/S at three Pi levels.

Source of variation	DF	Adjusted mean square		
		RApase	LApase	R/S ratio
Genotype	279	0.255***	1.526***	0.009***
Pi level	2	71.779***	252.245***	3.708***
Environment/experiment	1	0.029	0.004	0.0024
Genotype×Pi level	558	0.232***	1.056***	0.007***
Genotype×Environment/experiment	279	0.012	0.017	0.00228
Pi level×Environment/experiment	2	0.025	0.004	0.00136
Error	558	0.012	0.018	0.00277

*Significance at p< 0.05, **Significance at p<0.01 and ***Significance at p<0.001

**Table 2:** Descriptive statistics of RApase, LApase and R/S ratio at three Pi levels.

Pi level	Variables	Mean ± SE	Range	StDev	CV (%)
	RApase	1.06 ± 0.03	0.15-2.2	0.5	47.11
LP	LApase	2.18 ± 0.05	0.05-4.31	0.88	40.10
	R/S	0.25 ± 0.004	0.08-0.55	0.08	29.43
NP	RApase	0.35 ± 0.012	0.05-0.83	0.19	55.34
	LApse	1.94 ± 0.06	0.03-4.24	0.93	47.78
	R/S	0.12 ± 0.002	0.04-0.22	0.04	32.14
	RApase	0.75 ± 0.02	0.05-1.14	0.27	35.65
HP	LApase	0.92 ± 0.03	0.02-1.93	0.44	48.23
	R/S	0.11 ± 0.002	0.05-0.23	0.04	30.83

**Table 3:** Correlation analysis of BLUP estimated traits at three Pi levels.

Trait 1	Trait 2	LP (5 µM)	NP (250 µM)	HP (1 mM)
LApase	RApase	-0.057	0.05	-0.14*
R/S	RApase	-0.016	-0.074	-0.014
R/S	LApase	0.14*	-0.026	0.074

* Significance at p< 0.05.

### 3.2 Principal component analysis and GWAS for Apase activity

**Figure 3:**
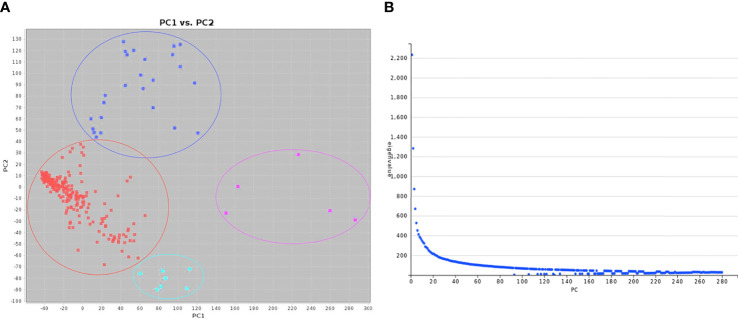
Principal component analysis of the diversity fixed foundation set of *Brassica juncea*: **(A)** Principal components and **(B)** Scree plot.

**Figure 4:**
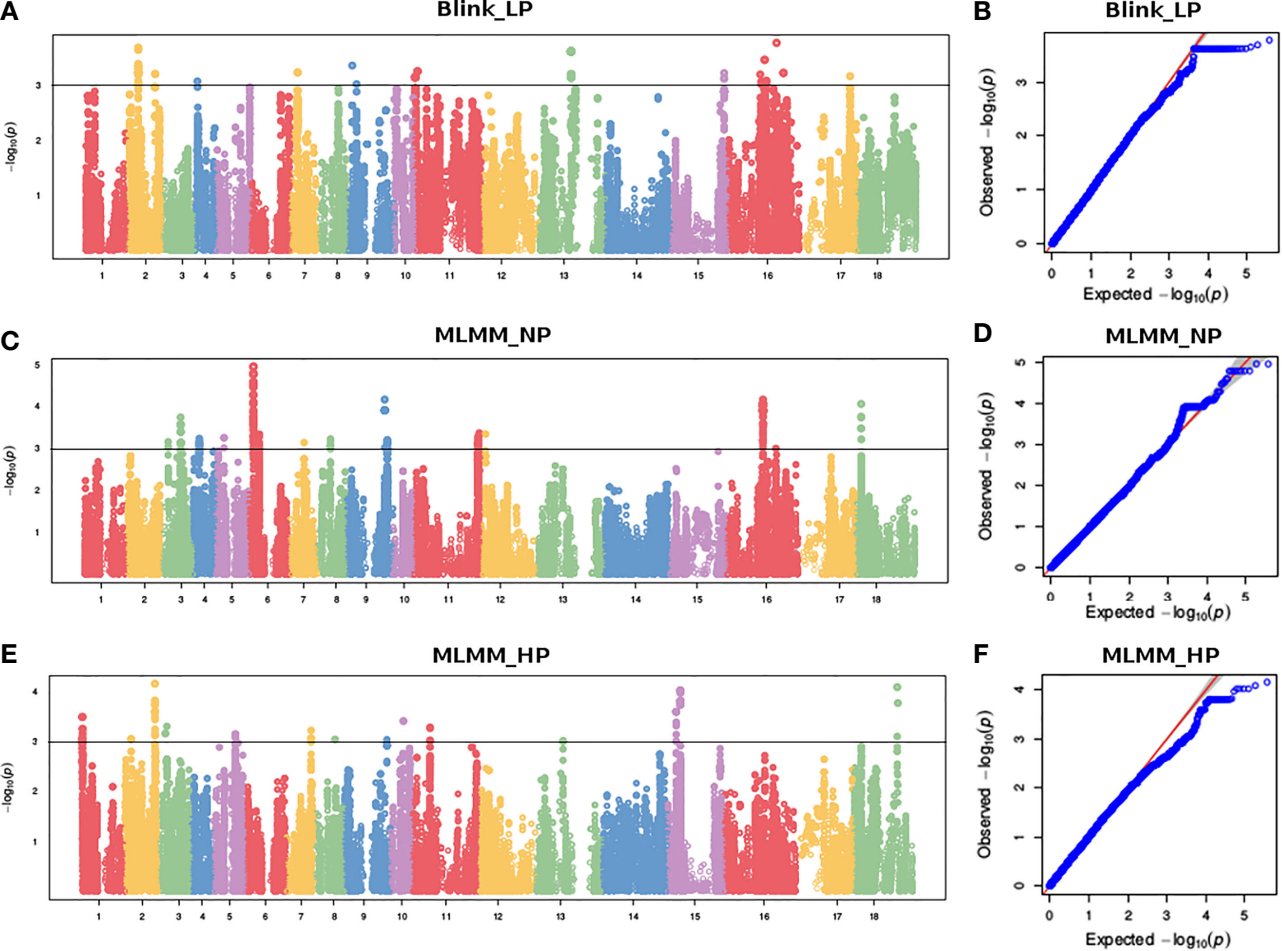
Manhatton and Q-Q plots showing marker trait associations for RApase at **(A, B)** LP, **(C, D)** NP and **(E, F)** HP levels.

**Figure 5:**
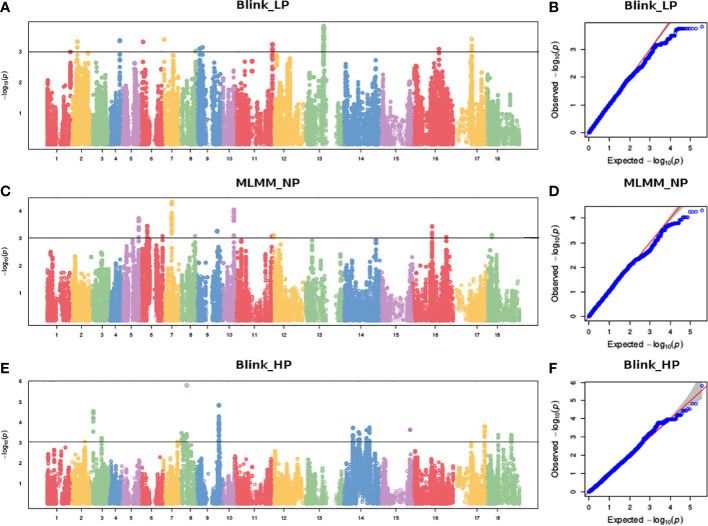
Manhatton and Q-Q plots showing marker trait associations for LApase at **(A, B)** LP, **(C, D)** NP and **(E, F)** HP levels.

To reduce false positives due to population structure, we used principal components (2 PCs) and kinship matrix as covariates in GWAS analysis. Two PCs depicted clear separation among populations (**Figure 3**). In total, four groups appeared. Groups I and II were less diverse than groups III and IV. Group I (shown in turquoise blue color) comprised only 5 genotypes. Groups II (magenta) and III (royal blue) included seven and twenty-five genotypes, respectively. Group IV (red color) was the largest group, with 243 genotypes. Groups I and II primarily comprised of exotic mustard genotypes. Most of the introgression and advanced breeding lines were in group III. Group IV was the mix of varieties, resynthesized genotypes and exotic *B. juncea.* GWAS was conducted to identify MTAs for RApase and LApase under LP, NP and HP levels (**Table 4**). A total of 3, 72,285 quality SNPs were used as marker dataset. The GAPIT3 package (Wang and Zhang, 2021) implemented with five algorithms (GLM, MLM, Farm CPU, MLMM and Blink) in R software was run for trait-SNP association analysis. An ideal model is supposed to show a fair degree of correspondence between the observed and expected p-values in the quantile–quantile (QQ) plots. We compared p values [observed − log10 (p-value)] and their expected ranked values [expected − log10 (p-value)] through Q-Q plots to test the predictability of applied GWAS models, over all environments. MLMM and BLINK were identified as the best fit models over all environments (**Supplementary Figure 1**). They showed minimum deviations from uniform distribution in multiple Q-Q plots. The estimated Bonferroni threshold value was 6.87. However, the value was found to be highly stringent to detect MTAs for a complex trait like Apase activity which might be controlled by several genes of minor effects. So, we used an arbitrary threshold value of -log 10 (p) ≥ 3.0 to identify MTAs for Apase activity. Manhattan plots depicting the associated SNPs for Apase activity in root and leaf tissues under three environments (LP, NP and HP) are presented in **Figures 4** and **5**. Associated SNPs and surrounding genomic regions were further annotated to decipher trait related genes. A total of 14 genomic regions involving 44 unique MTAs were envisioned for Apase activity (including both RApase and LApase) under LP, NP and HP levels on chromosomes A01, A03, A04, A05, A08, A09, B05, B06, B07 and B08 of *B. juncea* (**Table 4**). Ten associated regions were predicted for A genome chromosomes, while four for B genome chromosomes. Chromosome A01 revealed the maximum number of MTAs (21 SNPs). The identified QTLs accounted for 4.15 to 7.21% of phenotypic variation. Among 14, 10 regions were recorded for RApase and 4 for LApase. The number of QTLs detected explicitly under LP, NP and HP were 6, 3 and 6, respectively. We could not find any common MTA for Apase activity across three Pi doses. Functional annotation of 100 kbp region (50 kbp on both sides) of peak-associated SNPs, facilitated the identification of 15 candidate genes with diverse roles in the molecular regulation of Apase activity. Four genes encode diverse family members of Apases. Other genes have roles in phytohormone or sugar signalling pathways and root modulation. Genes *PAP10* and *PAP16*, which belong to PAP family (central family of Apase) of Apase, were predicted in association with RApase and LApase activities, respectively, under the NP environment. Also, genes, *AT5G51260* (HAD superfamily gene) and *PNP* (*polyribonucleotide nucleotidyltransferase* family gene), encoding Apases, were envisaged 21.48 and 27.84 kbp away from the peak SNPs B06_33902675 and A05_33349252, respectively, influencing RApase under LP condition. In the present study, gene *ARF5* (*AUXIN RESPONSE FACTOR5)* was visualized 4.83 kbp away from the SNP B07_55242497 governing LApase activity at the HP level. Gene *ABR1 (APETALA2 like ABA repressor 1*), associated with RApase activity at LP, was envisaged 36.31kbp away from the peak SNP A09_5080851. We annotated genes *PLC2, PLAIVA* and P*LAIVC*, encoding phospholipases that hydrolyze phospholipids and release secondary messengers for phytohormone signalling. At LP dose, the gene *PLC2* was predicted for the genomic region B05_52982160-52982181 associated with RApase. Seven SNPs (A01_874995-876161) were present near *PLAIVA* and P*LAIVC* depicting 7% variation for RApase under HP condition. Another important gene *HXK1* (*HEXOKINASE1*) involved in the sugar signalling pathway, was located 15.48 Kbp away from peak SNP A09_6643946. This gene explained 7% of the phenotypic variation for LApase activity under Pi deficit condition. Four genes (*RGF1, BRXL1, SHR*, and *SAUR 41*) controlling root architecture were also recorded in the close surroundings of associated regions. Under Pi sufficient conditions, a SNP (A03_ 20686760) on A03 was linked with a signalling peptide *RGF1* (*ROOT GROWTH FACTOR1*) controlling variation for RApase. Gene *BRXL1* (*BREVIS RADIX LIKE 1*) was predicted near the cluster of six SNPs (A04_18578091-18578642), influencing LApase activity in Pi deficit condition. Gene *CIPK6* (*CBL-interacting protein kinase 6*) was present in the vicinity of two SNPs (A08_19805050, A08_19805060) located on the A08 chromosome. *CIPK6* codes for a CBL (Calcineurin B-like proteins)-interacting protein kinase with role in Pi deficiency and ABA signalling pathways.

**Table 4:** Summary of marker trait associations identified for RApase and LApase at three Pi levels.

Trait	Pi level	Chr	Position and number of SNPs	Candidate Gene	Distance from peak SNP(Kb)	-log10(p)	R^2^
RApase	LP	A05	33349252	*PNP(AT3G03710)*	27.84	3.27	4.47
		A09	5080851	*ABR1(AT5G64750)*	36.31	3.36	4.61
		B05	52982160-52982181(3)	*PLC2(AT3G08510)*	47.15	3.16	4.31
		B06	33902675	*AT5G51260* *(HAD* superfamily*)*	21.48	3.05	4.15
	NP	A03	20686760	*RGF1(AT5G60810)*	33.93	3.46	6.35
		B08	4817280-4817319(3)	*PAP10(AT2G16430)*	38.60	3.48	6.37
	HP	A01	874995-876161(7)	*PLAIVA(AT4G37070)*	12.25	3.23	7.00
				*PLAIVC(AT4G37050)*	9.00	3.23	7.00
		A01	651481-655735(14)	*SHR(AT4G37650)*	26.91	3.21	7.00
		A08	19805050, 19805060(2)	*CIPK6(AT4G30960)*	31.63	3.04	7.00
		A09	43265797	*SAUR41(AT1G16510)*	35.82	3.04	7.00
LApase	LP	A04	18578091-18578642(6)	*BRXL1(AT2G35600)*	49.26	3.35	7.21
		A09	6643946-6643948(2)	*HXK1(AT4G29130)*	15.48	3.09	7.00
	NP	A05	30105013	*PAP16(AT3G10150)*	21.21	3.15	5.00
	HP	B07	55242497	*ARF5(AT1G19850)*	4.83	3.18	6.13

## 4 Discussion

Breeding for enhanced PUE in Indian mustard is imperative for yield increment at low production costs. The Apase class of enzymes is crucial to improve PUE, as their activity is largely associated to P remobilization within the plant and acquisition from the soil by hydrolyzing P rich organic compounds (Duff et al., 1994; Bhadouria and Giri, 2022). There are two groups of APases depending upon their site of action-extracellular (secreted; SAPs) and intracellular (IAPs) Apases (Tian et al., 2012; Tian and Liao, 2015). SAPs act upon external organic P complexes in rhizosphere to liberate Pi, whereas IAPs are involved in Pi recycling from internal P reservoirs of plant cells (Sanchez-Calderon et al., 2010; Chiou and Lin, 2011; Swetha and Padmavathi, 2016). Some Apases possess both SAP and IAP properties (Robinson et al., 2012; Deng et al., 2020). Additionally, Apase enzymes are revealed to regulate diverse plant processes like seed development, flowering, senescence, carbon metabolism, response to biotic and abiotic stresses, cellular signalling pathways, symbiotic association, and root development. In the present study, we studied the genetics of Apase activity using GWAS methodology on a diversity set in *B.juncea*. Enzyme activity was estimated in two plant tissues (leaf and root) at three doses of Pi application in a hydroponic system. Significant differences were observed for both LApase (Apase activity in leaf) and RApase (Apase activity in root) over three Pi levels. The mean Apase activity increased in both tissues with a decrease in Pi input that emphasized the enzyme involvement under Pi deprived condition. This was in correspondence with previous reports in Indian mustard, common bean crops and rapeseed (Haran et al., 2000; Yan et al., 2001; Zhang et al., 2010). However, at all Pi levels, LApase activity was higher than RApase. This indicted the greater or earlier response of LApase than RApase to Pi supply. Zhang et al. (2010) has studied the contributions of root secreted Apase and leaf intracellular APase to PUE in *B. napus* and reported a significant contribution of leaf Apase activity towards PUE whereas root secreted Apase has revealed no direct correlation with PUE. Intracellular Apases are believed to be synthesized prior to the secreted Apases under Pi deprived condition (Bozzo et al., 2004; Bozzo et al., 2006). Apase activity in leaf enables the plant to remobilize Pi from P rich biomolecules present in older tissues (Duff et al., 1994; Garcia et al., 2004; Zhang et al., 2010). In our study, LApase was observed with a significant positive correlation with R/S at LP level. This may be due to the additional role of Apase in modulating root architecture by induction of Pi signalling pathways (Wang et al., 2018; Cai et al., 2021). At only Pi sufficiency, RApase was negatively correlated to LApase. It indicated the differential response of Apase activity in root and leaf to Pi status.

We identified several candidate genes (15) on chromosomes (A01, A03, A04, A05, A08, A09, B05, B06, B07 and B08) that might affect Apase activity in the evaluated genotypes of *B. juncea*. Important among them were: *PAP10, PAP16, PNP* and *AT5G51260*, which encode different family members of Apases. In our study, *PNP* and *AT5G51260* were observed for RApase activity under LP environment, whereas, *PAP10* and *PAP16* governed the variation at NP dose for RApase and LApase, respectively. Overexpression of PAP10 (that shows both SAP and IAP properties) in Arabidopsis and rice have been found to significantly increase the plant’s ability to degrade Po and tolerance to low Pi stress (Wang et al., 2011; Deng et al., 2020). *PNP* and *AT5G51260* regulate Pi tolerance by releasing Pi during polynucleotide synthesis from nucleotide diphosphates or triphosphates (Marchive et al., 2010; Deng et al., 2021). We also predicted several genes with roles in phytohormone and sugar signalling pathways and root modulation. *ARF5*, a gene located 4.8 kbps away from the SNP B07_55242497, explained variation in Apase activity in leaf under Pi sufficient conditions. It codes for an auxin response factor 5 which transcriptionally regulates almost one-half of Aux/IAA genes (Krogan et al., 2014). Another auxin induced gene *SAUR 41*(*SMALL AUXIN UP RNA41*), appeared close to SNP A09_43265797. It is responsible for developing auxin-related phenotypes including root meristem repatterning in Arabidopsis (Kong et al., 2013). Under low Pi conditions, the gene *ABR1* (*APETALA2 like ABA repressor 1*) encodes a negative regulator of ABA-regulated gene expression (Pandey et al., 2005). A SNP B05_52982181 was linked to a phosphatidylinositol-specific phospholipase C2 encoded by gene *PLC2* for RApase activity at low Pi. This gene shows hydrolytic activity against phosphoinositides to release secondary messengers, myo-inositol-1,4,5-trisphosphate (InsP3) and diacylglycerol (DAG) that are known to improve plant tolerance against various types of biotic and abiotic stresses (Nokhrina et al., 2014). Another two patatin-related phospholipase A genes (*PLAIVA and PLAIVC*) were also predicted for RApase activity but at the high Pi level. They hydrolyze phospholipids and galactolipids and generate free fatty acids and lysolipids as secondary messengers that participate in phytohormone signalling for root development under normal and Pi stress conditions (Rietz et al., 2010). *HXK1* gene encoding *HEXOKINASE 1*, the first enzyme of glycolysis, which converts glucose into glucose 6-phosphate. Besides this phosphorylation activity, it mediates sugar signalling pathway to influence pant architecture (Barbier et al., 2021). Here, this gene explained 7% variation for LApase activity at LP level. For RApase activity at high Pi level, SNP A01_55242497 corresponded to gene *CIPK6*. This gene (*CIPK6*) codes for a CBL (Calcineurin B-like proteins) interacting protein kinase that functions in Pi starvation signalling pathway to enhance the plant tolerance to low Pi stress (Chen et al., 2012). Gene *BRXL1* associated with LApase activity under Pi deficit condition in the present study, determines the extent of cell proliferation and elongation in the plant roots (Mouchel et al., 2006; Beuchat et al., 2010). The SHR gene, which controls 7% of the variation in RApase at HP, plays an important role in the specification and maintenance of the root stem-cell niche (Niu et al., 2015). Gene *RGF1* encoding a signalling peptide is known to influence the circumferential cell number in the root meristem in response to low Pi environment (Cederholm and Benfey, 2015). We could associate this gene with RApase activity at NP dose. The present investigation is the first attempt at genome wide association mapping for Apase enzyme activity in root and leaf tissues of Indian mustard. Predicted genes could serve as potential targets for improving PUE in *Brassica juncea*, after due validation.

## 5 Conclusion

In this study, 280 mustard genotypes were examined for root and leaf Apase activities in two environments under three doses of Pi. GWAS analysis revealed a total of 44 SNPs significantly associated with two traits at three Pi levels. Functional annotation of genomic regions in or around SNPs facilitated the prediction of genes encoding diverse Apase family members, root modulators, signalling peptides, phytohormone-induced factors, and secondary messenger releasing enzymes. These findings provided useful information to improve the PUE of Indian mustard by marker-assisted selection in the future.

## Data availability statement

The data presented in the study are deposited in the Indian Biological Data Center (http://ibdc.rcb.res.in/) repository, accession number INRP000037.

## Author contributions

GK designed and supervised the whole experiment. PU performed phenotypic evaluations. RS and VS helped in conducting the experiment under hydroponic condition. SS assisted in the biochemical analysis. PU compiled the results and performed the statistical analysis. JA and SKS performed bioinformatics. PU, SKS and MG carried out annotation and wrote the manuscript. All authors have read and approved the published version of the manuscript.
