# Supplementary figures and images for: Genome wide association studies for acid phosphatase activity at varying phosphorous levels in Brassica juncea L
Source: Front Plant Sci. 2022 Dec 20;13:1056028. doi: 10.3389/fpls.2022.1056028 (PMC9808407; doi:10.3389/fpls.2022.1056028)

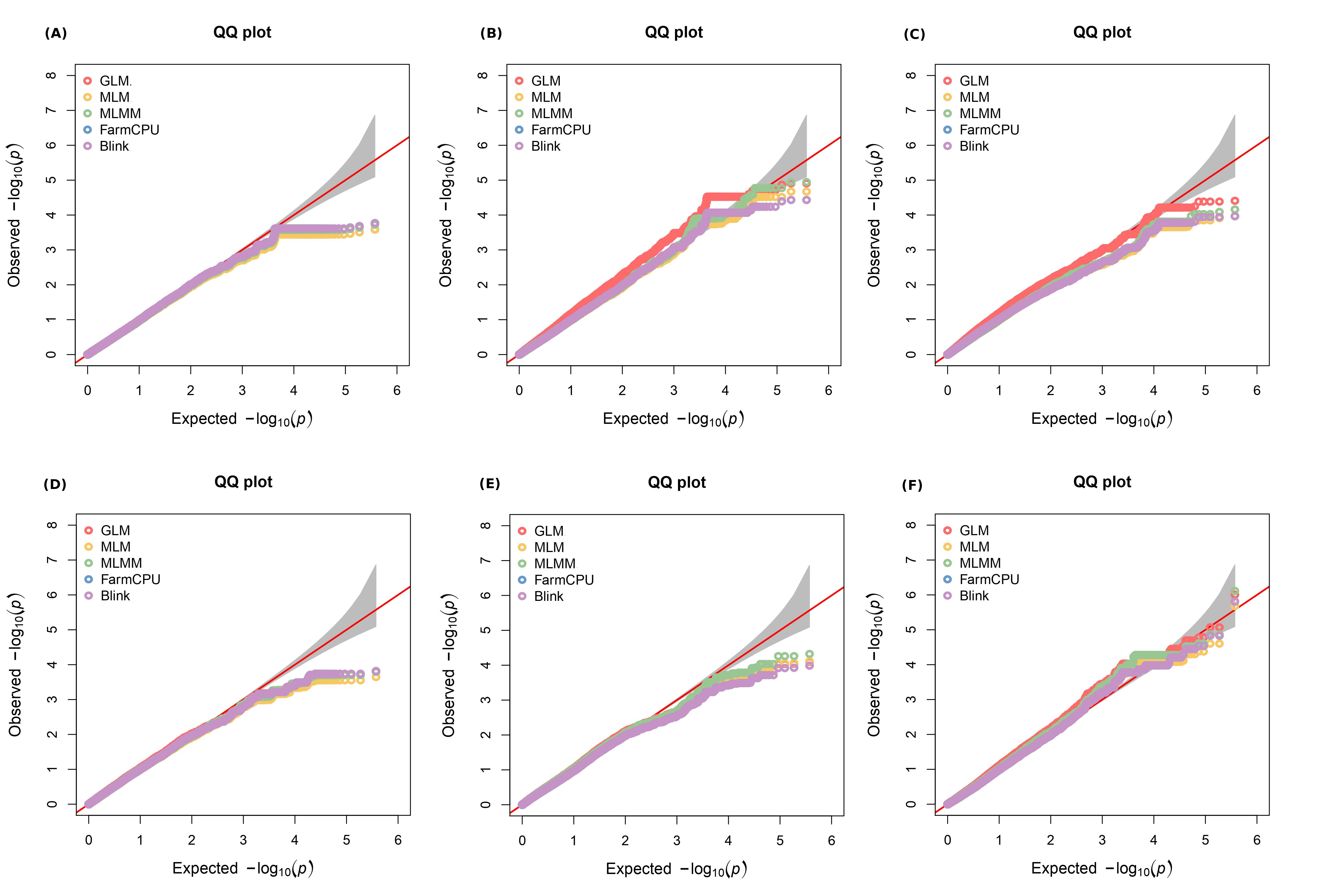

Supplement: Supplementary file 1 [file Image_1.tiff]
